# Microwave-assisted photooxidation of sulfoxides

**DOI:** 10.1038/s41598-021-99322-9

**Published:** 2021-10-21

**Authors:** Yuta Matsukawa, Atsuya Muranaka, Tomotaka Murayama, Masanobu Uchiyama, Hikaru Takaya, Yoichi M. A. Yamada

**Affiliations:** 1 RIKEN Center for Sustainable Resource Science, Wako, Saitama 351-0198 Japan; 2 Graduate School of Pharmaceutical Sciences, Nagoya University, Nagoya, 464-8601 Japan; 3 Cluster for Pioneering Research (CPR), Advanced Elements Chemistry Laboratory, RIKEN, Wako, Saitama 351-0198 Japan; 4 Graduate School of Pharmaceutical Sciences, The University of Tokyo, Bunkyo, Tokyo 113-0033 Japan; 5 Institute of Chemical Research, Kyoto University, Uji, Kyoto 611-0011 Japan

**Keywords:** Chemistry, Catalysis, Organic chemistry

## Abstract

We demonstrated microwave-assisted photooxidation of sulfoxides to the corresponding sulfones using ethynylbenzene as a photosensitizer. Efficiency of the photooxidation was higher under microwave irradiation than under conventional thermal heating conditions. Under the conditions, ethynylbenzene promoted the oxidation more efficiently than conventional photosensitizers benzophenone, anthracene, and rose bengal. Ethynylbenzene, whose T_1_ state is extremely resistant to intersystem crossing to the ground state, was suitable to this reaction because spectroscopic and related reported studies suggested that this non-thermal effect was caused by elongating lifetime of the T_1_ state by microwave. This is the first study in which ethynylbenzene is used as a photosensitizer in a microwave-assisted photoreaction.

## Introduction

Microwave (MW)-assisted reactions have recently been established as a successful synthetic method because of their enhanced reaction rates, increased yields, and suppressed side reactions and because they do not require solvents, unlike the thermally assisted reactions^1–18^. Non-thermal effects of MWs have been observed in some cases where the reaction proceeds much faster under MW irradiation than under conventional heating conditions. Interestingly, existence of the non-thermal effects of MWs is still a debatable topic^4,5^. The non-thermal effects of MWs are usually thought to originate due to three factors: dipolar polarization, ionic conduction, and non-thermal effects of highly polarizing radiation. Lately, the non-thermal effects of MWs were investigated at the liquid–solid interface, and nonequilibrium heating and accelerated electron transfer were determined to be the main factors responsible this effect^19,20^. This effect was also observed during decarboxylation using a silicon nanowire-array stabilized-Rh nanoparticle catalyst, and the reaction proceeded only under MW irradiation^8^. However, the precise mechanism by which these non-thermal effects are manifested is still elusive, and in particular, MW-assisted photoreactions are unexplored^21,22^. Considering this, we chose to investigate the photooxidation of sulfoxides in this study because the sulfones produced in this reaction have broad applications in the industrial, healthcare, and pharmaceutical sectors in the form of polyphenylsulfones, dimethyl sulfone^23^, and some coxibs^24^, respectively. Sulfones are synthesized either via the oxidation of sulfides or sulfoxides with peroxides^25,26^ or hypervalent iodine^27^, or by coupling sulfonates with haloarenes^28^, or via photooxidation^29^. However, these methods require the use of heavy metals or a high-pressure mercury lamp, and this should be strictly avoided. Thus, development of an unprecedented MW-assisted photooxidation reaction of sulfones using an organic photosensitizer is desirable both in the industry and academia. Herein, we report the ethynylbenzene-catalyzed photooxidation of sulfoxides and the mechanism by which the non-thermal effects of MWs are manifested in this reaction, where microwave is used to promote the photooxidation effectively.

## Results and discussion

The photooxidation of dimethylsulfoxide (**1a**) to dimethylsulfone (**2a**) was investigated using various photosensitizers under white light (Xe lamp) or MW irradiation (8 W, 2.45 GHz) at 50 °C for 20 h (Table 1). The temperature in the MW-irradiated conditions was monitored with an IR thermometer from outside, and was confirmed to be comparable to that measured with an optical fiber thermometer. The temperature of the reaction mixture was maintained at 50 °C during the reaction time. Ethynylbenzene (**3**) acted as a photosensitizer and promoted the oxidation to give **2a** in 31% yield (Entry 1; for detail, see Supplementary Figs. S1–S3 online). To the best of our knowledge, this is the first example of an oxidation reaction using ethynylbenzene as a photocatalyst. Notably, thermal heating alone, in the absence of MW irradiation, lowered the yield to 15% (Entry 2), suggesting that the oxidation was accelerated under MW irradiation. However, benzoic acid, presumably derived from **3**, was detected in 4% and 18% yields (Entries 1 and 2) in the presence and absence, respectively, of MW irradiation. The reaction did not proceed at all in the dark (Entry 3) and afforded the product in 5% yield in the absence of a photosensitizer (Entry 4). The yield of **2a** decreased when common photosensitizers, such as, benzophenone, anthracene, and rose bengal were used (Entries 5–7). Para-iodo substituted ethynylbenzene rather decreased the yield (Entry 9) comparing with non-substituted one (Entry 1). In the absence of MW, the yields afforded by rose bengal and *p*-iodoethynylbenzene showed no significant difference from those under irradiation of MW respectively (Entries 8 and 10). Thus, **3** was the best photosensitizer for this MW-assisted photooxidation. The role of the functional group on **3** was investigated next. Diphenylacetylene and biphenyl gave **2a** in 11% and 10% yields, respectively (Entries 11 and 12), suggesting that the terminal alkynyl group was the crucial moiety in the photosensitizer. Benzoic acid, which is presumably a decomposed product of **3**, did not have any specific role, either individually or in combination with **3**, in this reaction (Entries 13 and 14). No peracids were detected under any of the reaction conditions, suggesting that the oxidation did not proceed via peracid intermediates. Lowering the light intensity (15 mW/cm^2^ at 450 nm) decreased the yield of **2a** to 19% (Entry 13). When the reaction was conducted in toluene under the conditions corresponding to Entry 1, **2a** was obtained in 25% yield, suggesting that toluene is applicable to this reaction (Entry 16). Increasing the reaction time to 48 h linearly increased the yield to 77% (Entry 17). In the absence of either MW or light irradiation, **2a** was obtained in 21% and 0% yields, respectively, after 48 h (Entries 18 and 19). This observation suggested the synergistic effect of MW and light in this reaction. Only 2% yield of **2a** was detected in the presence of a selective singlet oxygen quencher Co(acac)_3_ (Entry 20).

**Table 1:** Screening of photosensitizer in the MW-assisted photooxidation of dimethyl sulfoxide (**1a**).


Entry	PS^*a*^	MW	Yield of 2a (%)^*b*^	Recovery of 1a (%)^***b***^
1	PhC≡CH **3**	+	31	69
2	**3**	−	15	81
3^*d*^	**3**	+	0	100
4	None	+	5	91
5	Benzophenone	+	12	87
6	Anthracene	+	24	74
7	Rose bengal	+	3	94
8	Rose bengal	−	4	93
9	*p*-I-C_6_H_4_-C≡CH	+	3	94
10	*p*-I-C_6_H_4_-C≡CH	-	7	89
11	PhC≡CPh	+	11	86
12	Ph–Ph	+	10	90
13	PhCO_2_H	+	7	91
14	**3** + PhCO_2_H	+	30	67
15^***d***^	**3**	+	19	81
16^***e***^	**3**	+	25	75
17^f.^	**3**	+	77	22
18^f.^	**3**	−	21	75
19^***b****,****f***^	**3**	+	0	100
20^***g***^	**3**	+	2	98

Reaction conditions: **1a** (15 mmol) and photosensitizer (0.75 mmol) were used with O_2_ balloon under MW (8 W) irradiation and white light irradiation from Xe lamp (30 mW/cm^2^ at 450 nm) at 50 °C for 20 h.

^a^PS = photosensitizer.

^*b*^Determined using ^1^H NMR with 1,3,5-trimethoxybenzene as the internal standard.

^*c*^In the dark.

^*d*^White light (15 mW/cm^2^ at 450 nm).

^*e*^In toluene (1 mL).

^*f*^48 h.

^*g*^In the presence of Co(acac)_3_ (0.75 mmol).

**Figure 1:**
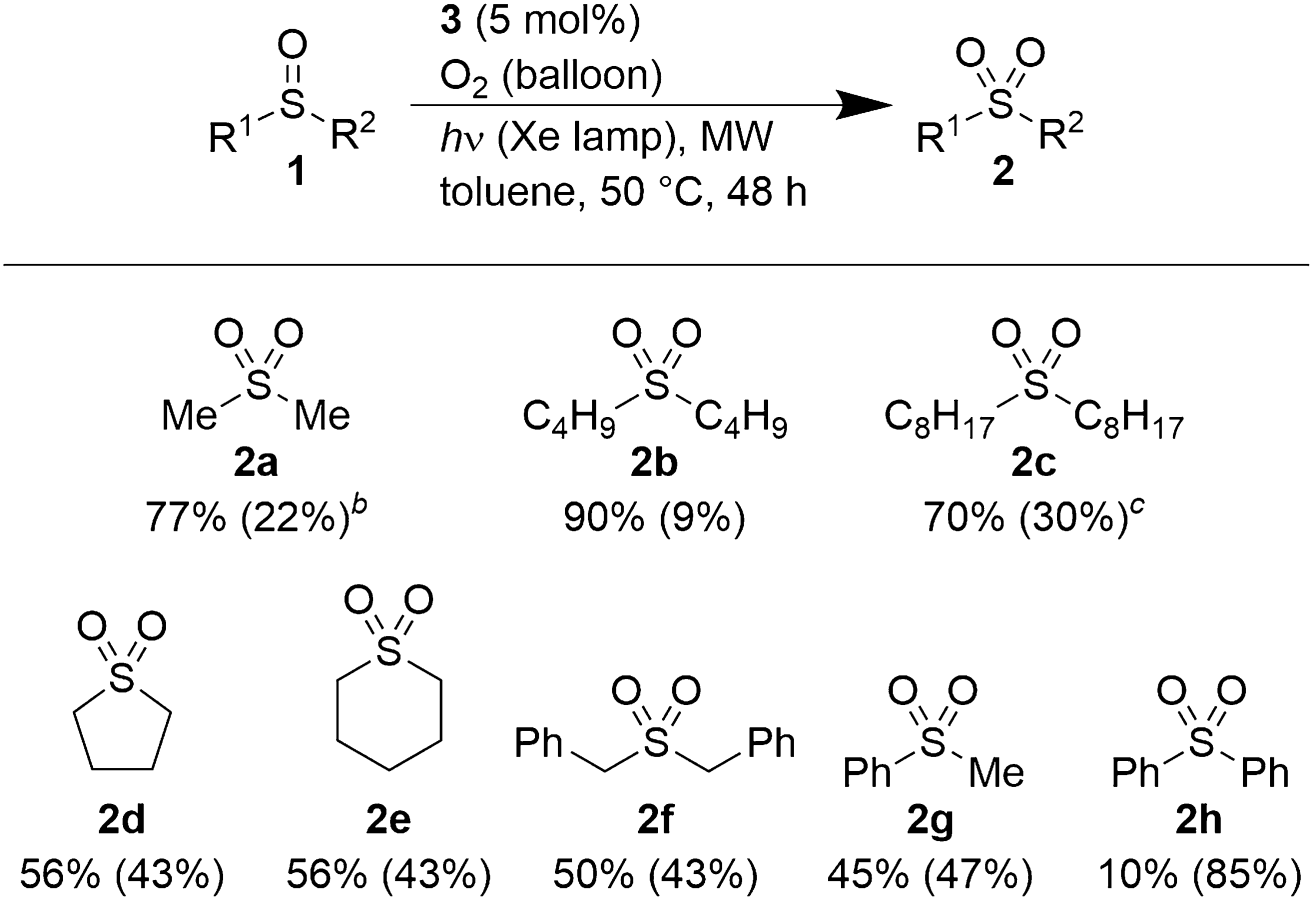
Substrate scope.^*a*^Reaction conditions: **1** (5.0 mmol) and **3** (0.25 mmol) were used in dry-toluene (2.0 mL) with O_2_ balloon under white light irradiation using a Xe lamp (30 mW/cm^2^ at 450 nm) and MW irradiation (8 W) at 50 °C. Yields were determined using ^1^H NMR with 1,3,5-trimethoxybenzene as the internal standard. The Numbers in parentheses mean the rate of substrate recovery. ^*a,b*^**1a** (15 mmol) and **3** (0.75 mmol) were used without any solvent. ^*c*^1.0 mL of dry-toluene was used.

With the optimized conditions in hand, various sulfoxides were examined for this reaction (Fig. 1) using toluene as a solvent. Compounds **1b** (dibutyl sulfoxide) and **1c** (dioctyl sulfoxide) were oxidized to give **2b** and **2c** in 90% and 70% yields, respectively. Cyclic sulfoxides **1d** and **1e** and dibenzyl sulfoxide **1f.** were also converted to the corresponding sulfones in 50–56% yields. Aromatic substrates like methyl phenyl sulfoxide (**1g**) and diphenyl sulfoxide (**1h**) were oxidized to give the corresponding products in 45% and 10% yields, respectively. Thus, alkyl sulfoxides are oxidized efficiently in this reaction, while the product yields decrease with increasing number of phenyl groups in the substrate. This further implies that the electron density of the sulfur atom and/or the competition between **3** and the phenyl groups for light absorption affect the yields. This is also true when **1a** or **1f.** Is used as the substrate. For substrates **1b–1h**, benzaldehyde was obtained as the byproduct and is probably derived from toluene.

**Figure 2:**
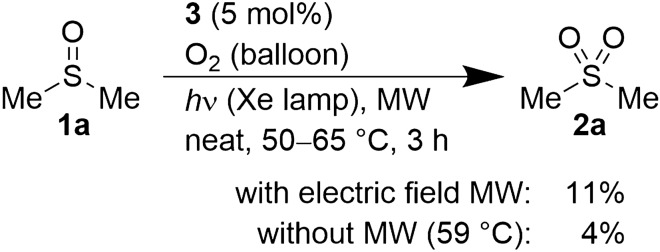
Effect of the electric field component of MW.

To gain insights into the mechanism, MW was irradiated as an individual electric field using a single-mode cavity (employing *E*-max in TM_010_ mode; for detail, see Supplementary Figs. S4–S5 online)^8,30–32^ for 3 h under the optimized conditions mentioned in Fig. 2. Compound **2a** was obtained in 11% yield in this mode, while it was obtained in 4% yield under thermal heating conditions. This suggested that the electric field component of the MW contributed to the progress of this reaction.

To investigate the reaction mechanism, electronic absorption spectrum of ethynylbenzene was acquired in DMSO. Ethynylbenzene showed an absorption band in the region 260–320 nm, which is beyond the absorption edge of DMSO (Supplementary Table S2 and Fig. S7a online). This indicated that the ethynylbenzene could be effectively photoexcited in DMSO. The absorption band of **3** in this region was assigned to the π–π* transition^33^. The π– π* transition corresponding to the S_0_-S_1_ transition was further confirmed by density functional theory at the B3LYP/6-31G* level (Supplementary Table S1 and Fig. S6 online).

**Figure 3:**
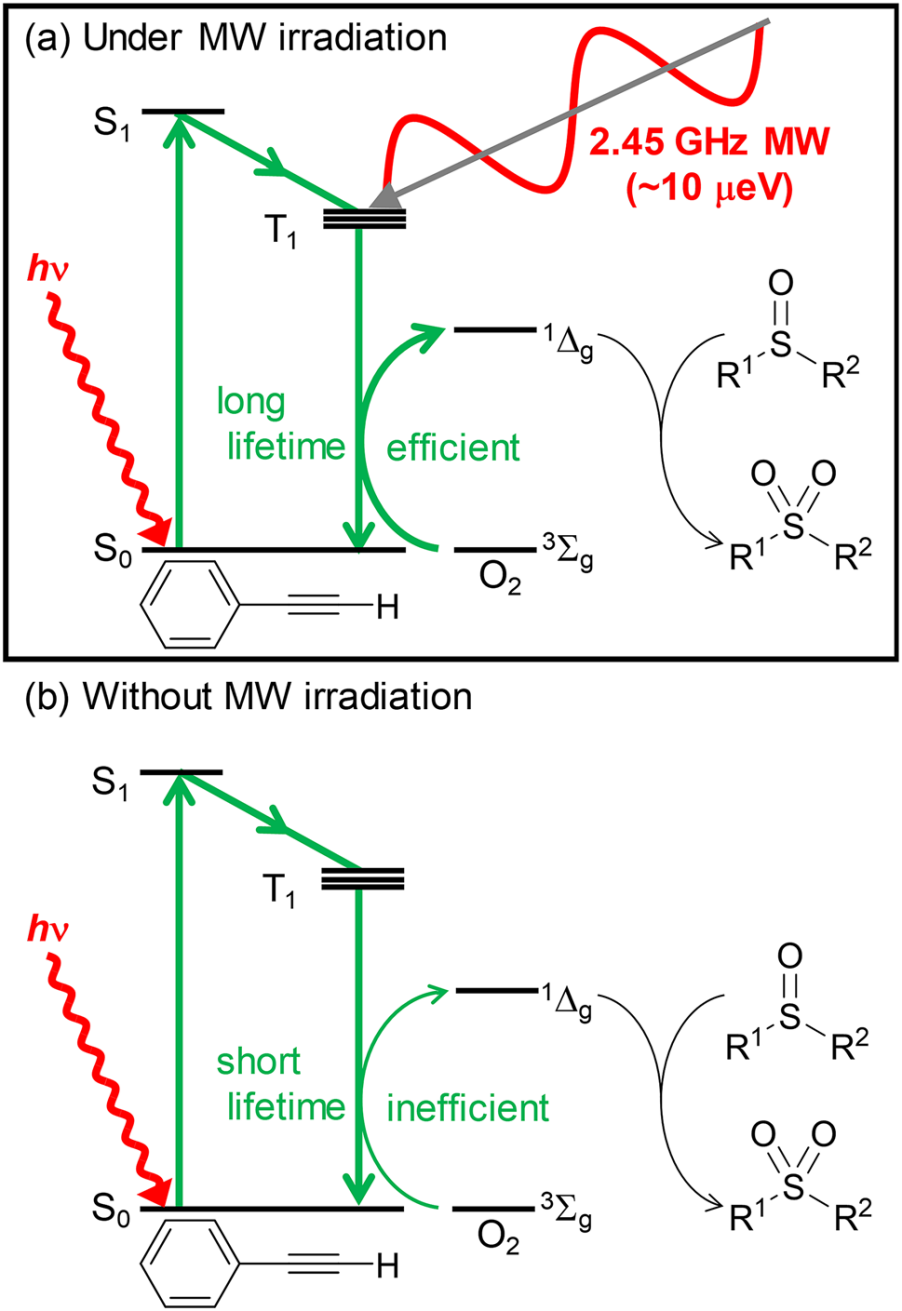
Proposed energy dissipation pathways. Green arrows indicate the direction of energy transfer as the oxidation progresses. Thickness of the arrows corresponds to the feasibility of the process. (**a**) Mechanism under MW irradiation using ethynylbenzene. (**b**) Energy dissipation pathways of ethynylbenzene and O_2_ in the absence of MW.

Based on the above observations, a plausible mechanism of oxidation was proposed (Fig. 3). Upon UV light absorption, **3** is excited from the S_0_ (singlet ground) state to the S_1_ (excited) state, which is converted to the T_1_ state through intersystem crossing. In the T_1_ state, **3** reacts with triplet dioxygen to excite the singlet oxygen, which subsequently oxidizes the sulfoxide, and then relaxes to the S_0_ state. The lifetime of the T_1_ state of ethynylbenzene is the key to the success of this reaction. Johnson and Sears reported that the T_1_ state of ethynylbenzene is extremely resistant to intersystem crossing to the ground state^34^. The significance of the T_1_ state seems to be reflected in the result shown in Table 1, Entry 9 as the phosphorescence lifetimes of the iodo-substituted ethynylbenzene, whose lifetimes decrease markedly with increasing mass of the iodine atom^35^. The results in control experiments using various halo substituted ethynylbenzenes also support this hypothesis (Table S1). Moreover, MW is known to make a significant perturbation to triplet spin state population with elongate its lifetime^36^. Photon energy of MW of 2.45 GHz frequency is ~10 μeV, and this is of the same order as the energy difference between the original T1 state and the two sublevels (9 and 12 μeV, calculated from the reported zero field parameters D and E)^37^. Such variation in population in the T_1_ state is known in the field of optically detected magnetic resonance (ODMR) spectroscopy^38^. The longer lifetime T_1_ state under the MW irradiation makes the encounter with O_2_ more efficient and thus, the oxidation proceeds more efficiently (Fig. 3a) compared to that involving the short-lived T_1_ state without irradiation of MW (Fig. 3b). The increased yield under MW irradiation can be attributed to this effect. The mechanism involving singlet oxygen was supported by a detection of singlet oxygen luminescence (21% quantum yield) in the presence of ethynylbenzene measured in toluene (Figure S16) and the result shown in Table 1, entry 20 (The measurements were carried out in toluene and in DMSO with benzophenone as the standard (quantum yield of singlet oxygen generation (Φ = 29%^39^). The measurement in toluene showed a luminescence signal of singlet oxygen, whose quantum yield was calculated to 21% (Figure S16), whereas that in DMSO, no signal was observed.).

## Summary

In summary, we developed the MW-assisted photo-induced auto-oxidation of sulfoxides using ethynylbenzene as a photosensitizer. The oxidation is initiated by the π–π* transition, and the electric field of the MW is considered to prolong the lifetime of the T_1_ state that catalyzes the subsequent reaction. This speculation is supported by the correlation between the yields obtained using the iodo-substituted ethynylbenzene and the atomic weight of the corresponding iodine. Extended lifetimes of the T_1_ state upon MW irradiation have been utilized only in ODMR spectroscopy, and thus, this study is expected to bridge this spectroscopic technique with organic reactions and pioneer a new interdisciplinary area. Further investigation by survey of ethynylbenzene derivatives will be undertaken for improving the conversion of the substrates.

## Methods

### General

A 10 mL quartz vial or 6 mL glass vial (NT-16H, purchased from the Maruemu Corporation) were used, which was placed in a MW reactor (Discover, purchased from CEM Japan Corporation, or MR-2G-200R, purchased from Ryowa-electronics Corporation) or in an aluminum block on a hot plate under an irradiation of white light emitted from a Xe lamp (purchased from Asahi spectra).

### General procedure for the oxidation using MW reactor

To a mixture of ethynylbenzene (0.75 mmol) and dry-DMSO **1a** (15 mmol) in the 10 mL quartz vial, quartz beads were added (3.0 mm, 7 pcs). The vial was filled with O_2_, and closed with a silicone rubber cap which was connected via a PTFE tube to an O_2_ balloon. The vial was placed in the MW reactor (Discover) equipped with a quartz rod which guides white light emitted from the Xe lamp to the reactor, and heated at 50 ºC using MW (8 W) under an irradiation of the white light (30 mW/cm^2^, fixed at 450 nm) with blowing with a compressor. After a certain reaction time, the resulting mixture was analyzed by ^1^H NMR (500 MHz, CDCl_3_) to calculate a yield of **2a** and a recovery of **1a** using 1,3,5-trimethoxybenzene (δ 3.77, 9H) as the internal standard. In the cases of substrates **1b–1f**, 5.0 mmol of a substrate in dry-toluene (**1b**: 1.0 mL, the others: 2.0 mL) was used instead of **1a** without the quartz beads.

### General procedure for the oxidation under conventional thermal heating conditions

The 6 mL glass vial containing a mixture of ethynylbenzene (0.75 mmol) and dry-DMSO (15 mmol) was filled with O_2_, and closed with a septum cap equipped with a quartz rod which guides white light emitted from the Xe lamp to the vial. To the vial, a needle which was connected via a silicone tube to an O_2_ balloon was inserted through the septum. The vial was placed in the aluminum block on a hot plate and heated at 50 °C under an irradiation of the white light (30 mW/cm^2^, fixed at 450 nm). After a certain reaction time, the resulting mixture was analyzed by ^1^H NMR (500 MHz, CDCl_3_) to calculate a yield of **2a** and a recovery of **1a** using 1,3,5-trimethoxybenzene (δ 3.77, 9H) as the internal standard.

### General procedure for the oxidation under an electric field mode of MW

To a mixture of ethynylbenzene (0.31 mmol) and dry-DMSO **1a** (6.1 mmol) in the 10 mL quartz vial, quartz beads were added (3.0 mm, 7 pcs). The vial was filled with O_2_, and closed with a silicone rubber cap which was connected via a PTFE tube to an O_2_ balloon. The vial was placed at the maximum points of the electric field in the MW single mode cavity equipped with an IR thermometer, a wave detector, a double-stub tuner connected to the MW generator (MR-2G-200R, 2.5 GHz), and a quartz rod which guides white light emitted from the Xe lamp to the reactor, and then heated at 50 ºC with MW (0.6 W) under an irradiation of the white light (30 mW/cm^2^, fixed at 450 nm) with blowing with DC fan (San-Ace, Sanyo Denki Corporation). After 3 h, the resulting mixture was analyzed by ^1^H NMR (500 MHz, CDCl_3_) to calculate a yield of **2a** and a recovery of **1a** using 1,3,5-trimethoxybenzene (δ 3.77, 9H) as the internal standard.

## Supplementary Information

Supplementary Information.
