# Supplementary material for: Microwave-assisted photooxidation of sulfoxides
Source: Sci Rep. 2021 Oct 21;11:20505. doi: 10.1038/s41598-021-99322-9 (PMC8531024; doi:10.1038/s41598-021-99322-9)
Supplement: Supplementary file 1 — Supplementary Information. [file 41598_2021_99322_MOESM1_ESM.pdf]

Microwave-assisted photooxidation of sulfoxides

Yuta MATSUKAWA,[a, b] Atsuya MURANAKA,[a,c] Tomotaka MURAYAMA,[c,d] Masanobu UCHIYAMA,[c,d] Hikaru TAKAYA,[e] Yoichi M. A. YAMADA*[a]

a. RIKEN Centre for Sustainable Resource Science, Wako, Saitama 351-0198, Japan.
E-mail: ymayamada@riken.jp

b. Graduate School of Pharmaceutical Sciences, Nagoya University, Nagoya, 464-8601, Japan.

c. Cluster for Pioneering Research (CPR), Advanced Elements Chemistry Laboratory, RIKEN, Wako, Saitama 351-0198, Japan.

d. Graduate School of Pharmaceutical Sciences, The University of Tokyo, Bunkyo, Tokyo 113-0033, Japan.

e. Institute of Chemical Research, Kyoto University, Uji, Kyoto, 611-0011, Japan.

1. General • • • S2

2. Experimental Section • • • S2

3. Control Experiments • • • S7

4. Calculated Absorption Properties of Ethynylbenzene • • • S8

5. Electronic Absorption Spectra of Ethynylbenzenes • • • S8

6. ^{1}H NMR Spectra of Oxidations of 1a–1h. • • • S10

7. Emission spectra of singlet oxygen in the presence of 3 • • • S14

8. Reference • • • S14

1. General

A 10 mL quartz vial or 6 mL glass vial (NT-16H, purchased from the Maruemu Corporation) were used, which was placed in a MW reactor (Discover, purchased from CEM Japan Corporation, or MR-2G-200R, purchased from Ryowa-electronics Corporation) or in an aluminum block on a hot plate under an irradiation of white light emitted from a Xe lamp (purchased from Asahi spectra). Emission spectra of singlet oxygen were recorded on a JASCO FP-6600 spectrofluorometer equipped with a Hamamatsu C9940 photomultiplier tube.

2. Experimental Section

General Procedure for the Oxidation Using MW Reactor. To a mixture of ethynylbenzene (0.75 mmol) and dry-DMSO **1a** (15 mmol) in the 10 mL quartz vial, quartz beads were added (3.0 mm, 7 pcs). The vial was filled with O_2, and closed with a silicone rubber cap which was connected via a PTFE tube to an O_2 balloon. The vial was placed in the MW reactor (Discover) equipped with a quartz rod which guides white light emitted from the Xe lamp to the reactor, and heated at 50 ºC using MW (8 W) under an irradiation of the white light (30 mW/cm^2, fixed at 450 nm) with blowing with a compressor. After a certain reaction time, the resulting mixture was analyzed by ^{1}H NMR (500 MHz, $CDCl_3$) to calculate a yield of **2a** and a recovery of **1a** using 1,3,5-trimethoxybenzene (δ 3.77, 9H) as the internal standard. In the cases of substrates **1b–1f**, 5.0 mmol of a substrate in dry-toluene (**1b**: 1.0 mL, the others: 2.0 mL) was used instead of **1a** without the quartz beads.

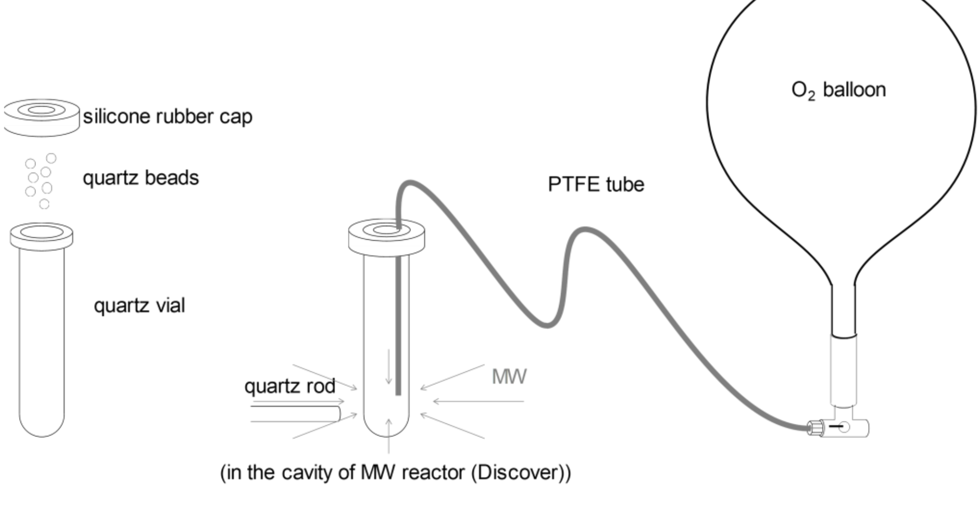

Figure S1. Schematic image of the experimental system in the presence of MW.

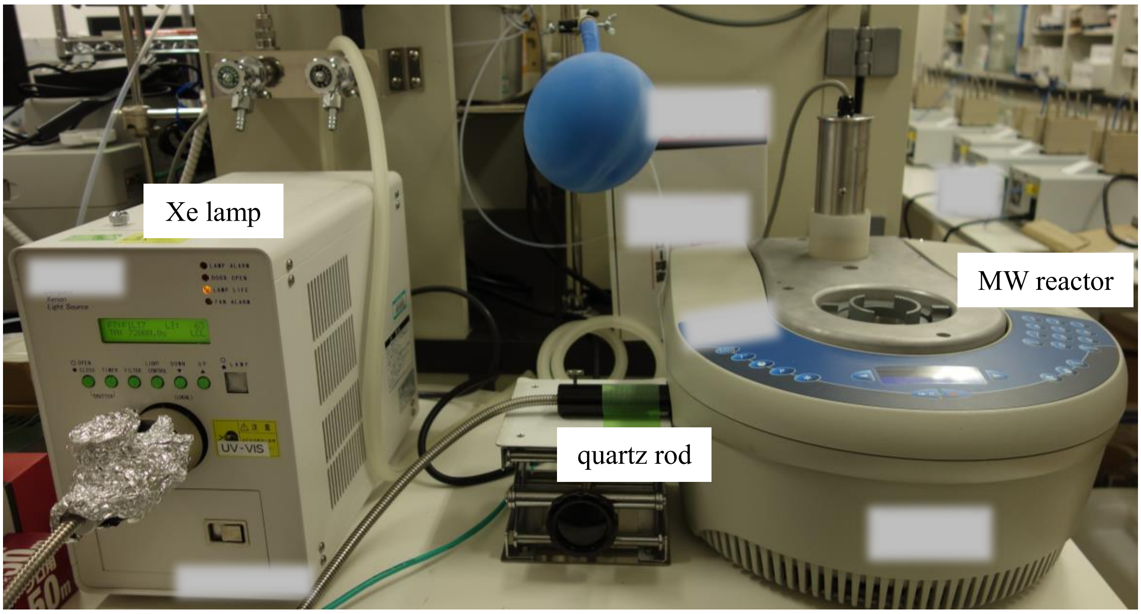

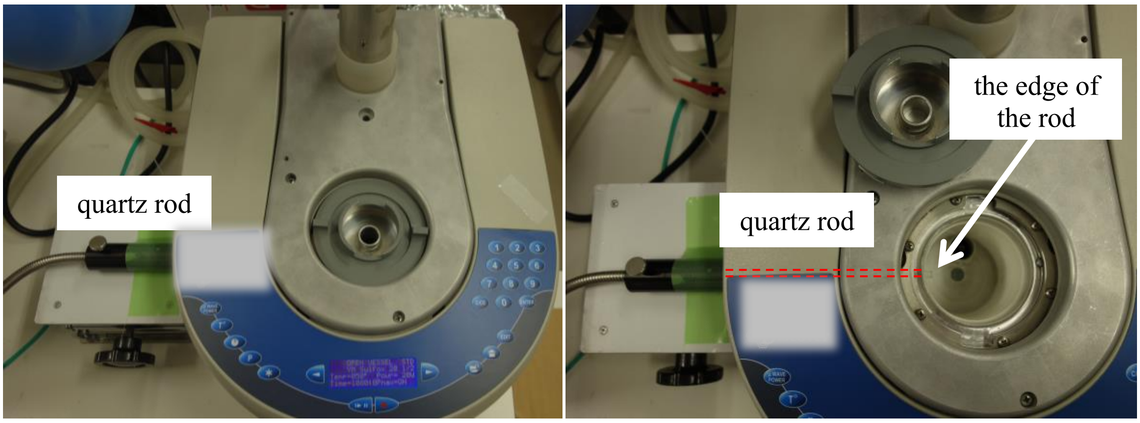

Figure S2. Pictures of the Xe lamp unit and the MW reactor (CEM Discover).

General Procedure for the Oxidation under Thermal Heating Conditions. The 6 mL glass vial containing a mixture of ethynylbenzene (0.75 mmol) and dry-DMSO (15 mmol) was filled with O_2, and closed with a septum cap equipped with a quartz rod which guides white light emitted from the Xe lamp to the vial. To the vial, a needle which was connected via a silicone tube to an O_2 balloon was inserted through the septum. The vial was placed in the aluminum block on a hot plate and heated at 50 ºC under an irradiation of the white light (30 mW/cm^2, fixed at 450 nm). After a certain reaction time, the resulting mixture was analyzed by ^{1}H NMR (500 MHz, $CDCl_3$) to calculate a yield of **2a** and a recovery of **1a** using 1,3,5-trimethoxybenzene (δ 3.77, 9H) as the internal standard.

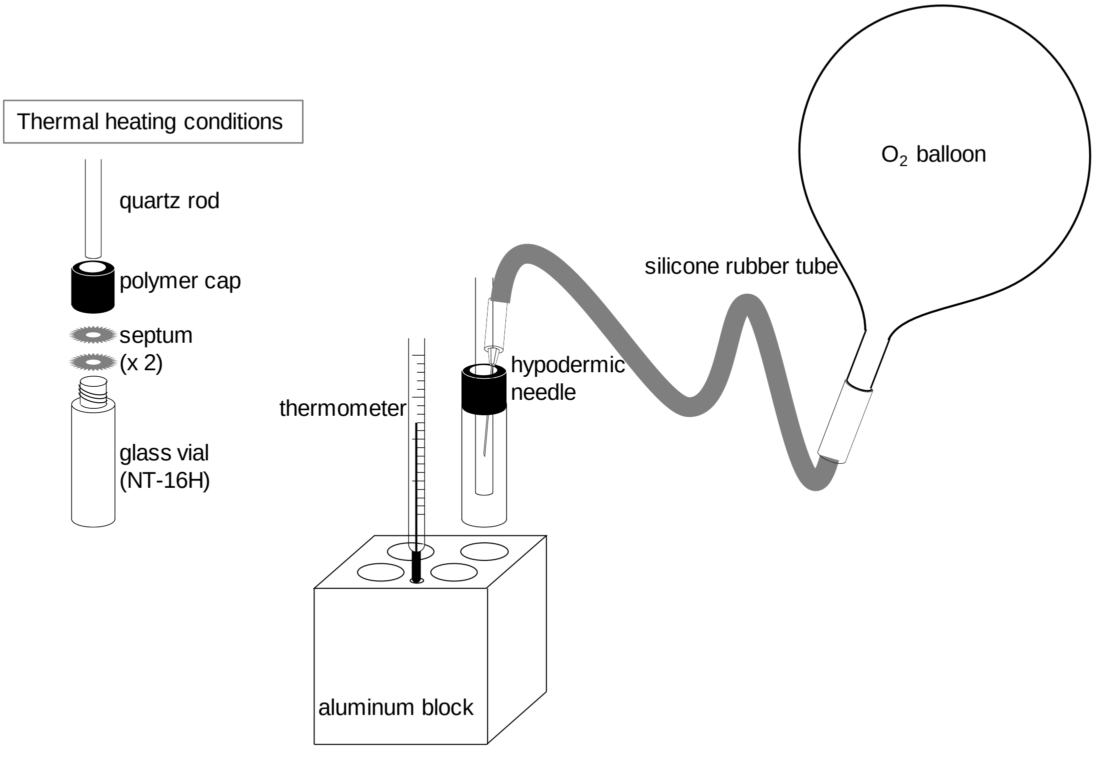

Figure S3. Schematic image of the experimental system under thermal heating.

General Procedure for the Oxidation under an Electric Field Mode of MW. To a mixture of ethynylbenzene (0.31 mmol) and dry-DMSO **1a** (6.1 mmol) in the 10 mL quartz vial, quartz beads were added (3.0 mm, 7 pcs). The vial was filled with O_2, and closed with a silicone rubber cap which was connected via a PTFE tube to an O_2 balloon. The vial was placed at the maximum points of the electric field in the MW single mode cavity equipped with an IR thermometer, a wave detector, a double-stub tuner connected to the MW generator (MR-2G-200R, 2.5 GHz), and a quartz rod which guides white light emitted from the Xe lamp to the reactor, and then heated at 50 ºC with MW (0.6 W) under an irradiation of the white light (30 mW/cm^2, fixed at 450 nm) with blowing with DC fan (San-Ace, Sanyo Denki Corporation). After 3 h, the resulting mixture was analyzed by ^{1}H NMR (500 MHz, $CDCl_3$) to calculate a yield of **2a** and a recovery of **1a** using 1,3,5-trimethoxybenzene (δ 3.77, 9H) as the internal standard.

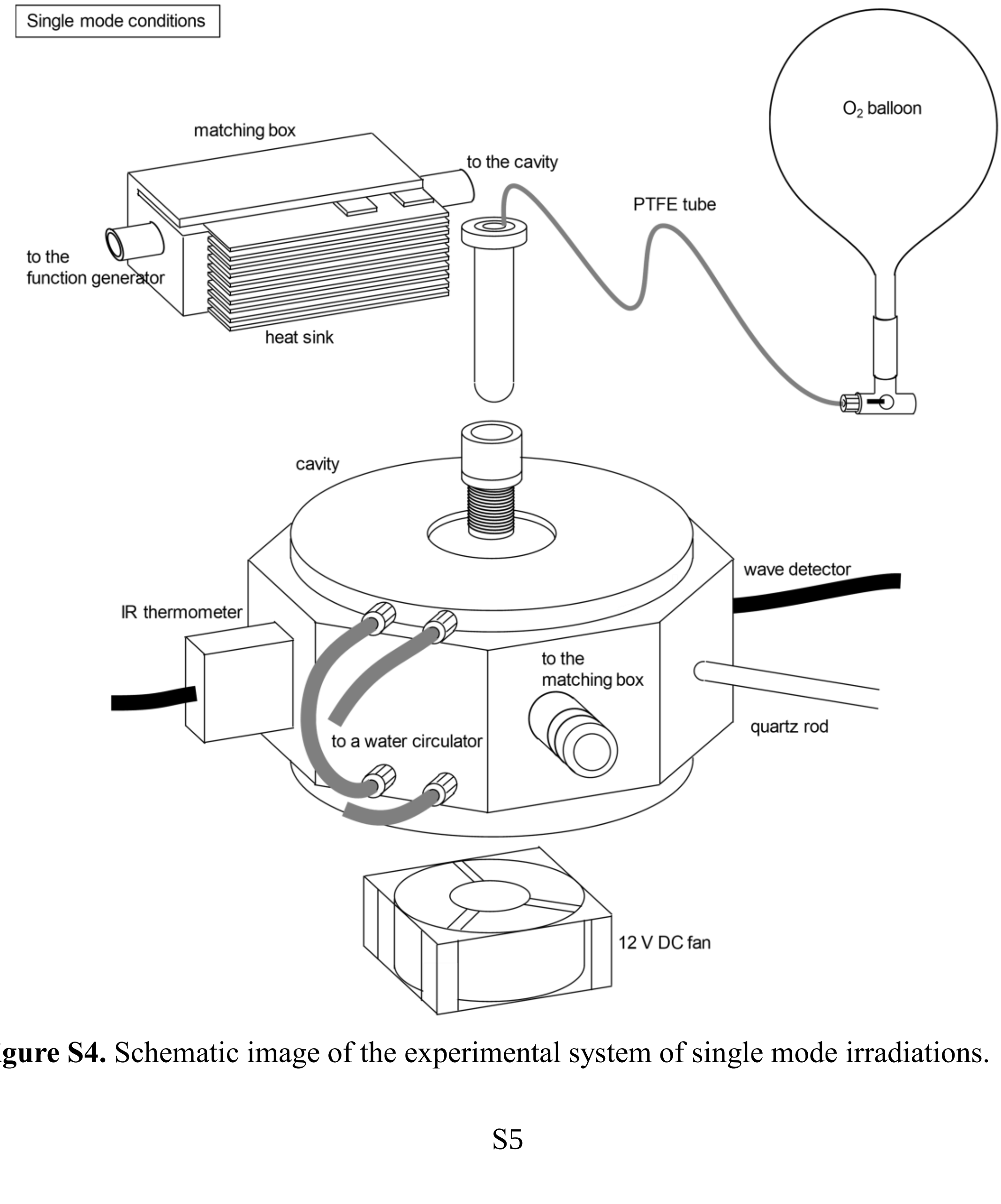

Figure S4. Schematic image of the experimental system of single mode irradiations.

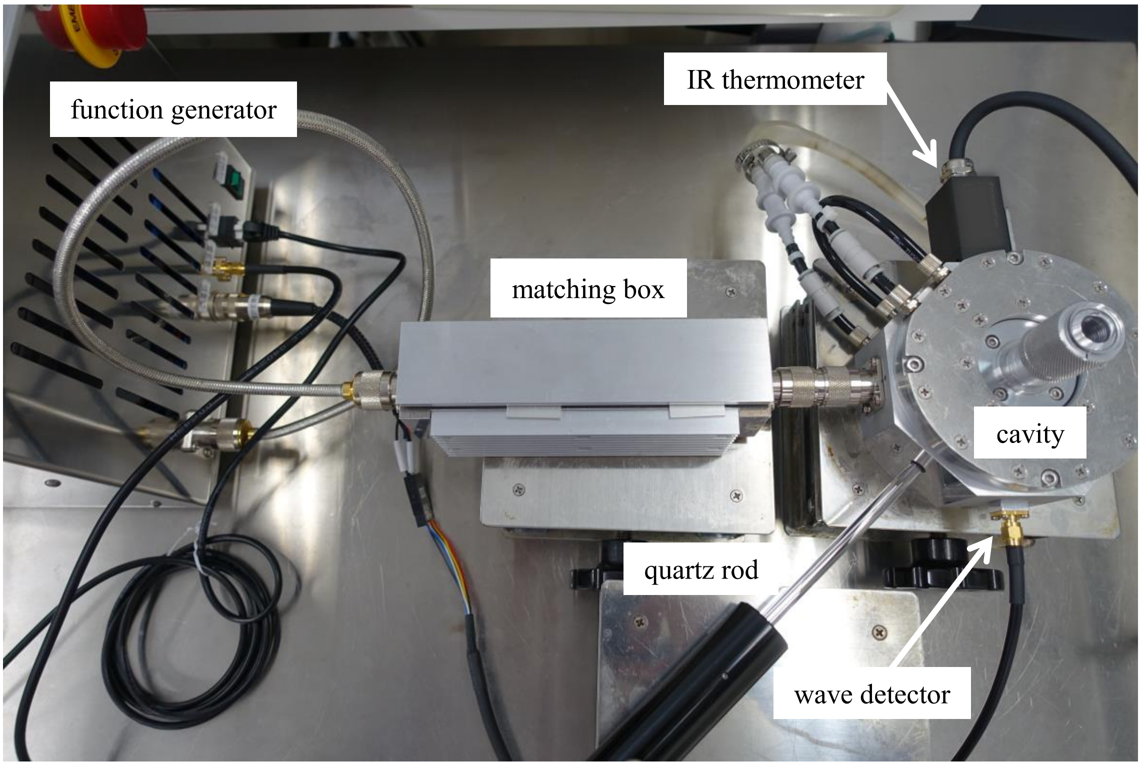

Figure S5. Pictures of the function generator, tuner, and the cavities for electric field mode (Ryowa MR-2G-200R).

Synthesis of Substrates. After cooling a solution of pentamethylene sulfide (3.06 g, 30.0 mmol) in CH_2Cl_2 (107 mL) to 3 ºC with ice bath, *m*-CPBA (contains 30 wt% of H_2O, 7.90 g, 32.0 mmol) was added in portion and the resulting mixture was stirred for 5.0 h. The mixture was washed with 2.50 M NaOH (15.0 mL, 37.5 mmol), and then dried over Na_2SO_4. The solvent was evaporated in vacuo to give a yellow liquid, which was purified on SiO_2 column chromatography (CH_2Cl_2 : MeOH = 95 : 5) to afford pentamethylene sulfoxide (**1e**, 2.41 g, 20.4 mmol, 60%, colorless liquid) along with sulfolane **2e** (346 mg, 2.58 mmol, 9%, pale brown crystals). The obtained **1e** was dried with CaH_2 at 68 ºC for 1.5 days followed by Kugelrohr distillation (110 ºC, 2 mbar) to give pure **1e** (1.86 g, 15.7 mmol, 46%, colorless crystals). ^{1}H NMR (500 MHz, $CDCl_3$): δ 1.55−1.71 (4H, m), 2.21−2.27 (2H, m), 2.74−2.79 (2H, m), 2.86−2.90 (2H, m). Dibenzyl sulfide (8.01 g, 37.4 mmol) was also oxidized likewise to afford dibenzyl sulfoxide (**1f**, 8.20 g, 35.6 mmol, 95%, colorless crystals) without any purification. ^{1}H NMR (500 MHz, $CDCl_3$): δ 3.88 (2H, d, J = 13 Hz, CH_2), 3.93 (2H, d, J = 13 Hz, CH_2), 7.29 (4H. d, J = 8.0 Hz, *o*-H), 7.34−7.40 (6H, m, *m*-H and *p*-H).

3. Control experiments

Table S1. Control experiments using halo-substituted ethynylbenzenes.[a]

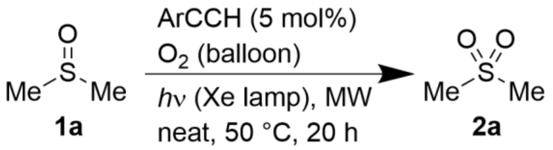

entry	ArC≡CH[b]	MW	Yield of **2a** (%)[b]	Recovery of **1a** (%)[b]
1	PhC≡CH **3**	+	31	69
2	*o*-Cl-C_6H_4-C≡CH	+	18	78
3	*m*-Cl-C_6H_4-C≡CH	+	32	65
4	*p*-Cl-C_6H_4-C≡CH	+	32	65
5	*p*-Cl-C_6H_4-C≡CH	-	11	88
6	*p*-Br-C_6H_4-C≡CH	+	27	70
7	*p*-Br-C_6H_4-C≡CH	-	19	81
8	*p*-I-C_6H_4-C≡CH	+	3	94
9	*p*-I-C_6H_4-C≡CH	-	7	89

[a]Reaction conditions: **1a** (15 mmol) and ArC≡CH (0.75 mmol) were used with O_2 balloon under irradiation of Xe lamp white light (30 mW/cm^2 at 450 nm) and MW (8 W) at 50 ºC for 20 h. [b]Determined by ^{1}H NMR with 1,3,5-trimethoxybenzene as the internal standard.

4. Calculated Absorption Properties of Ethynylbenzene

All calculations were performed at the Density Functional Theory (DFT), by means of the B3LYP functional level as implemented in Gaussian 16.[1] The 6-31G(d,p) basis set was used for each atom. The solvent effect (DMSO) was taken into account using the self-consistent reaction field (SCRF) method. Excitation wavelengths and oscillator strength for the optimized structure were calculated by the TD-DFT approach.

Table S2. Excitation wavelength (λ), oscillator strengths (f), and compositions for the S_0 – S_1 transition of ethynylbenzene at the level of B3LYP/6-31G(d,p).

λ [nm]	f	Composition (weight, %)[a]
245	0.0006	H–1 → L (50.6), H → L+1 (48.7)

[a] H = HOMO, L = LUMO.

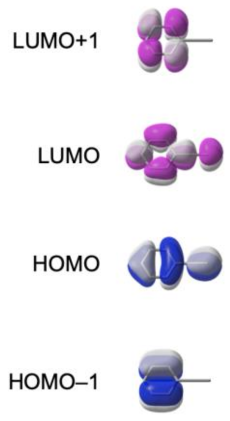

Figure S6. Selected frontier molecular orbitals of ethynylbenzene calculated at the level of B3LYP/6-31G(d,p).

5. Electronic Absorption Spectra of Ethynylbenzene

Table S3. Maximum absorption wavelength (λ) and molar extinction coefficient (ε) of ethynylbenzene measured in DMSO.

	λ_1	λ_2	λ_3	λ_4
λ [nm]	270.0	276.5	280.5	285.5
ε [$M^{-1} \cdot cm^{-1}$]	402	328	268	106

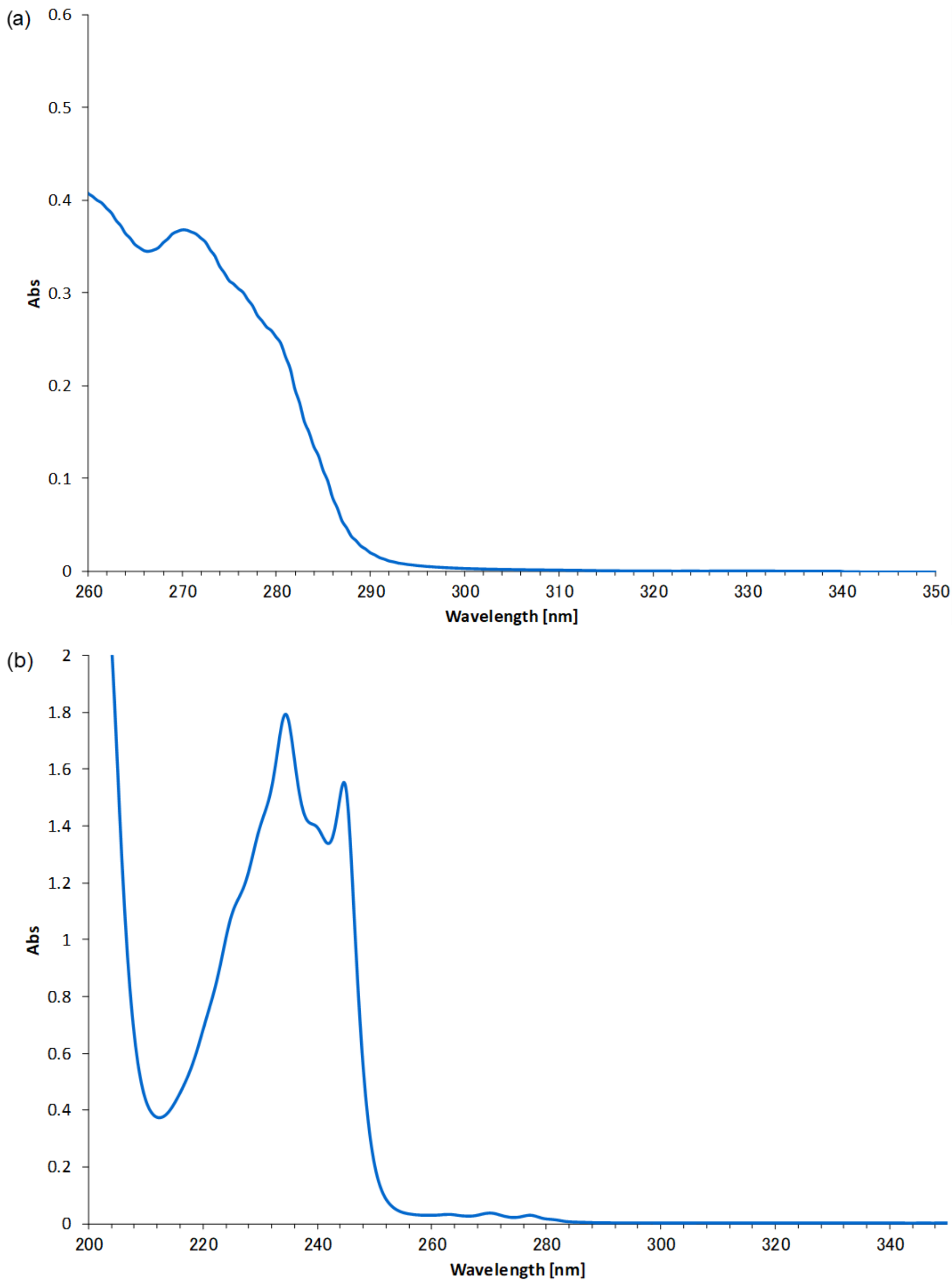

Figure S7. Electronic absorption spectra of ethynylbenzene (a) in DMSO and (b) in hexane.

6. ^{1}H NMR Spectra of Oxidations of 1a–1h.

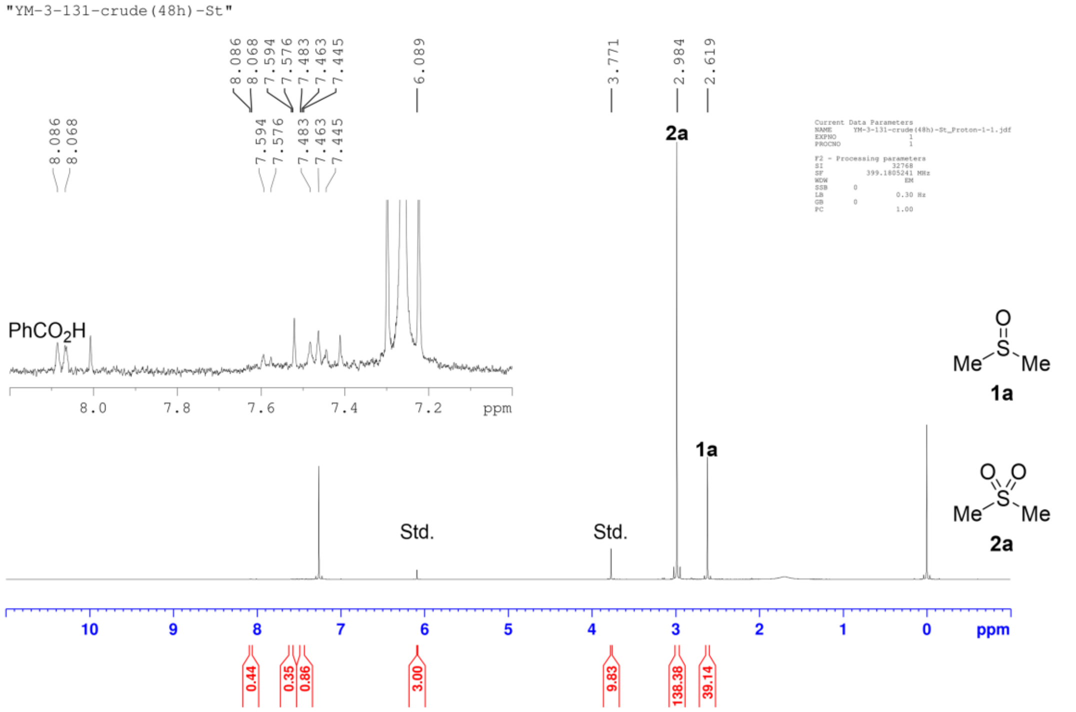

Figure S8. ^{1}H NMR spectra of an oxidation of **1a**.

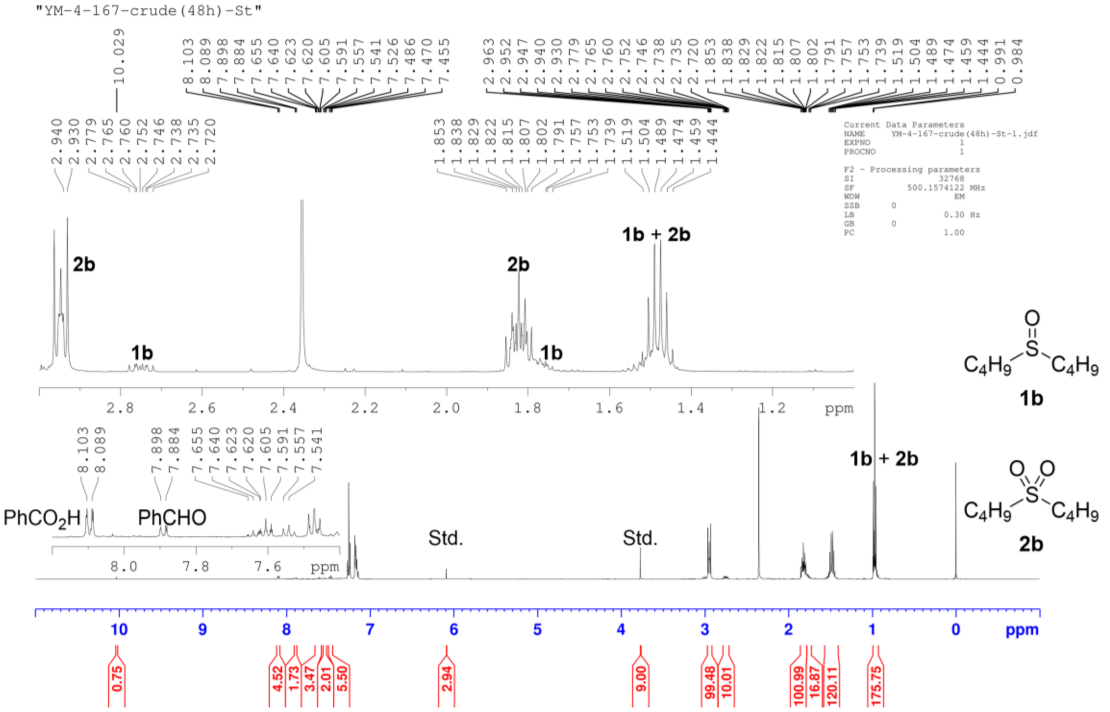

Figure S9. ^{1}H NMR spectra of an oxidation of **1b**.

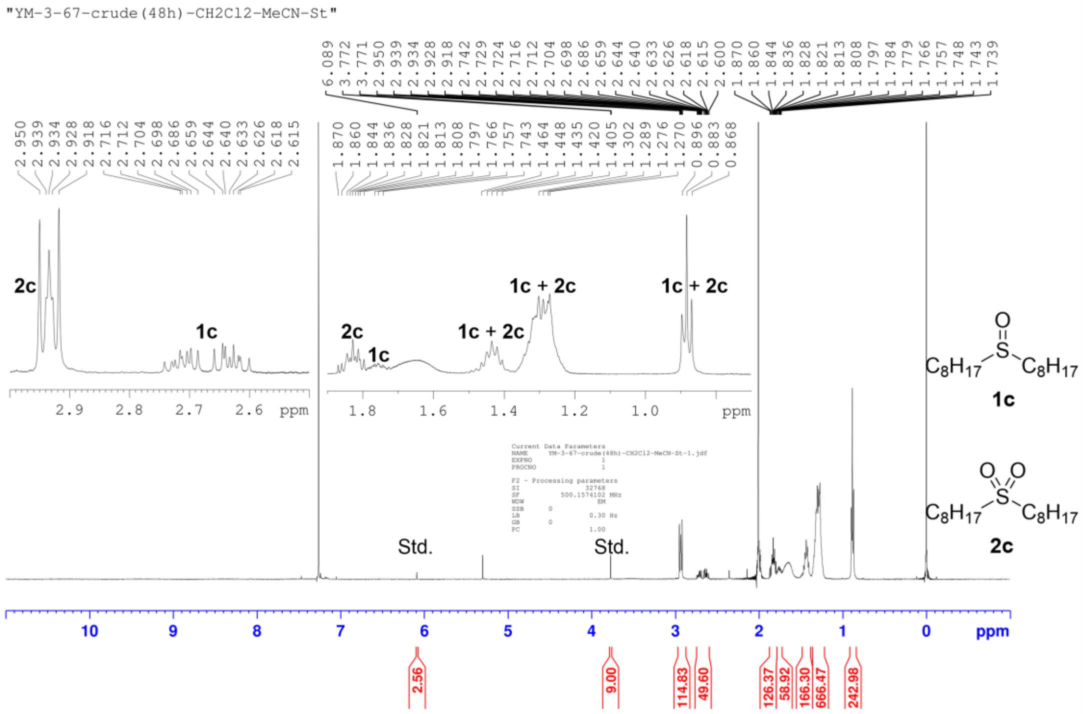

Figure S10. ^{1}H NMR spectra of an oxidation of **1c**.

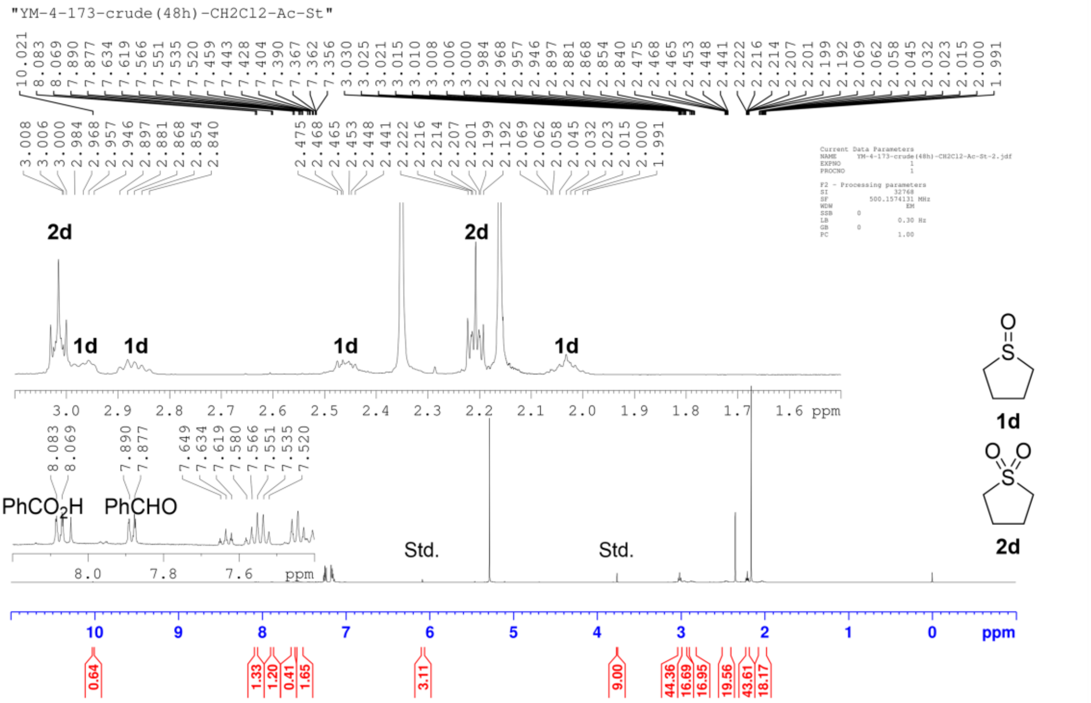

Figure S11. ^{1}H NMR spectra of an oxidation of **1d**.

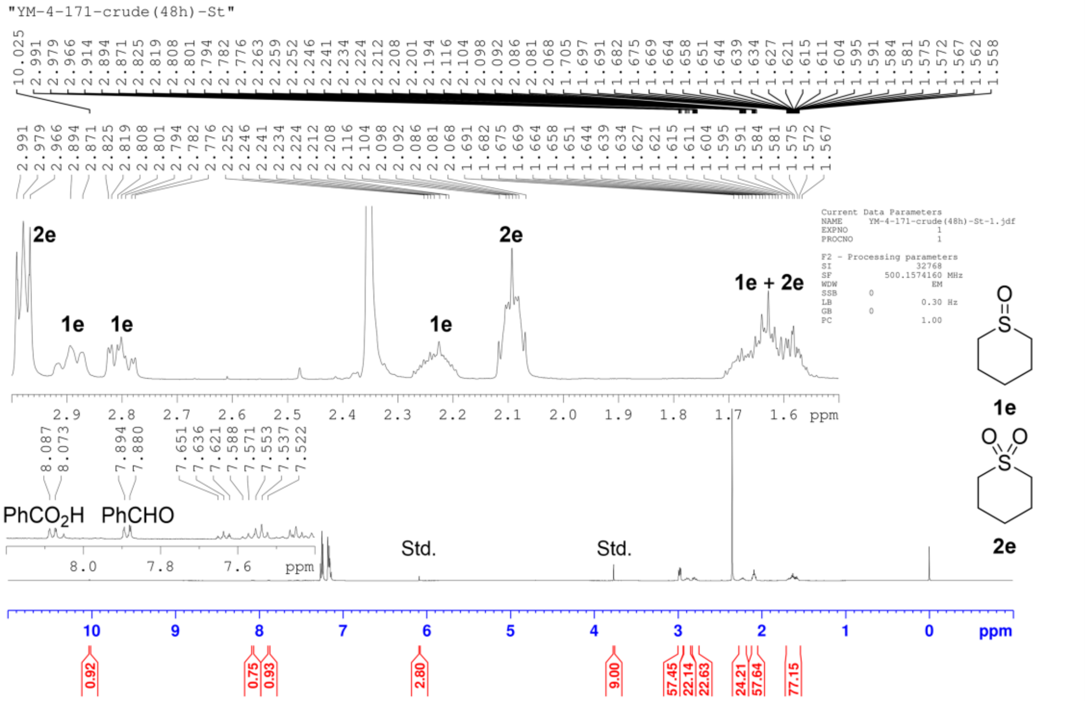

Figure S12. ^{1}H NMR spectra of an oxidation of **1e**.

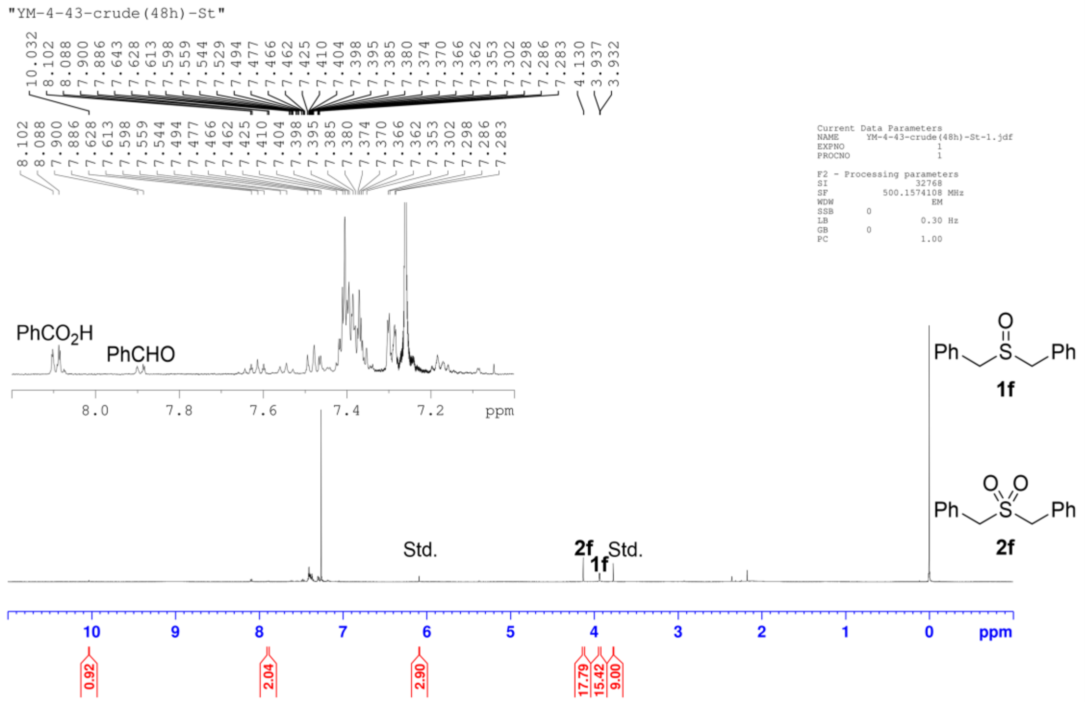

Figure S13. ^{1}H NMR spectra of an oxidation of **1f**.

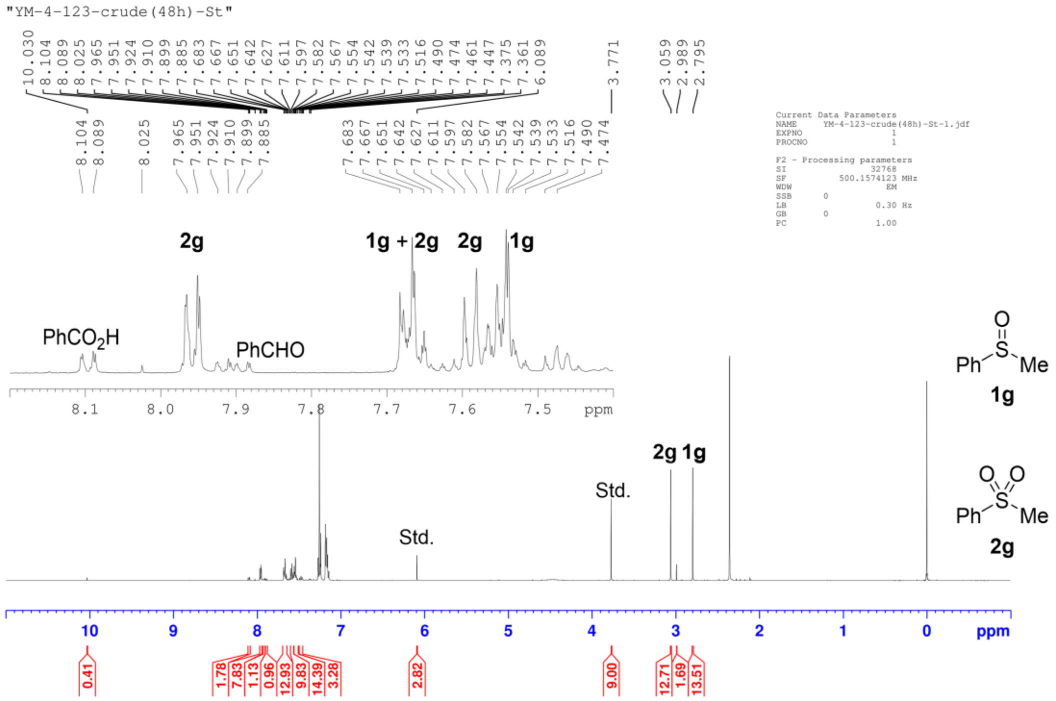

Figure S14. ^{1}H NMR spectra of an oxidation of **1g**.

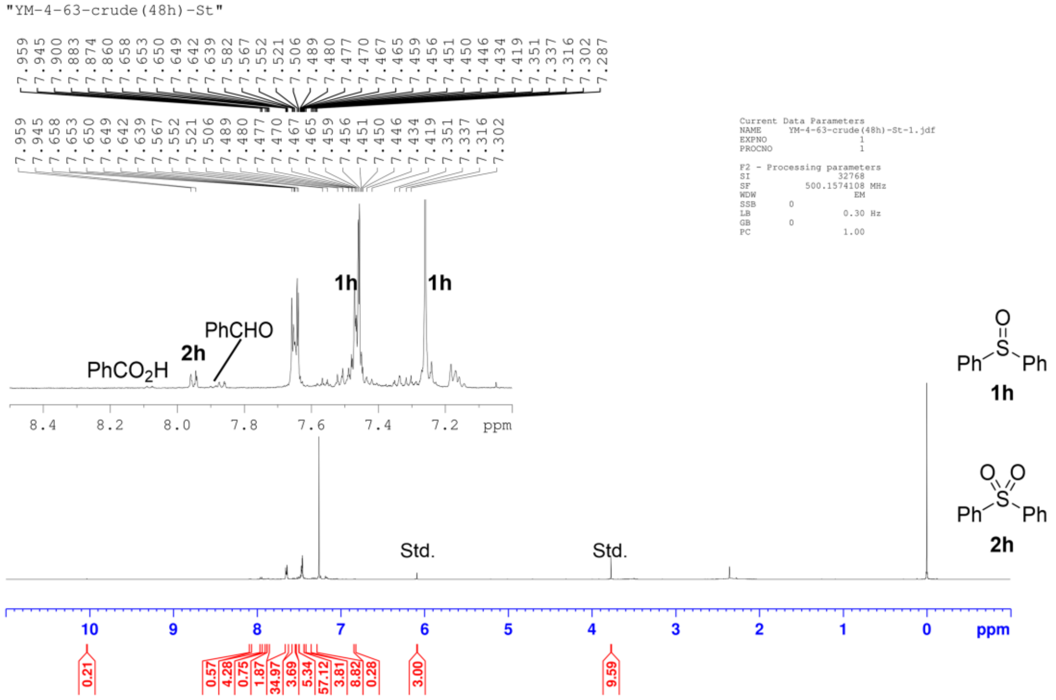

Figure S15. ^{1}H NMR spectra of an oxidation of **1h**.

7. Emission spectra of singlet oxygen in the presence of 3

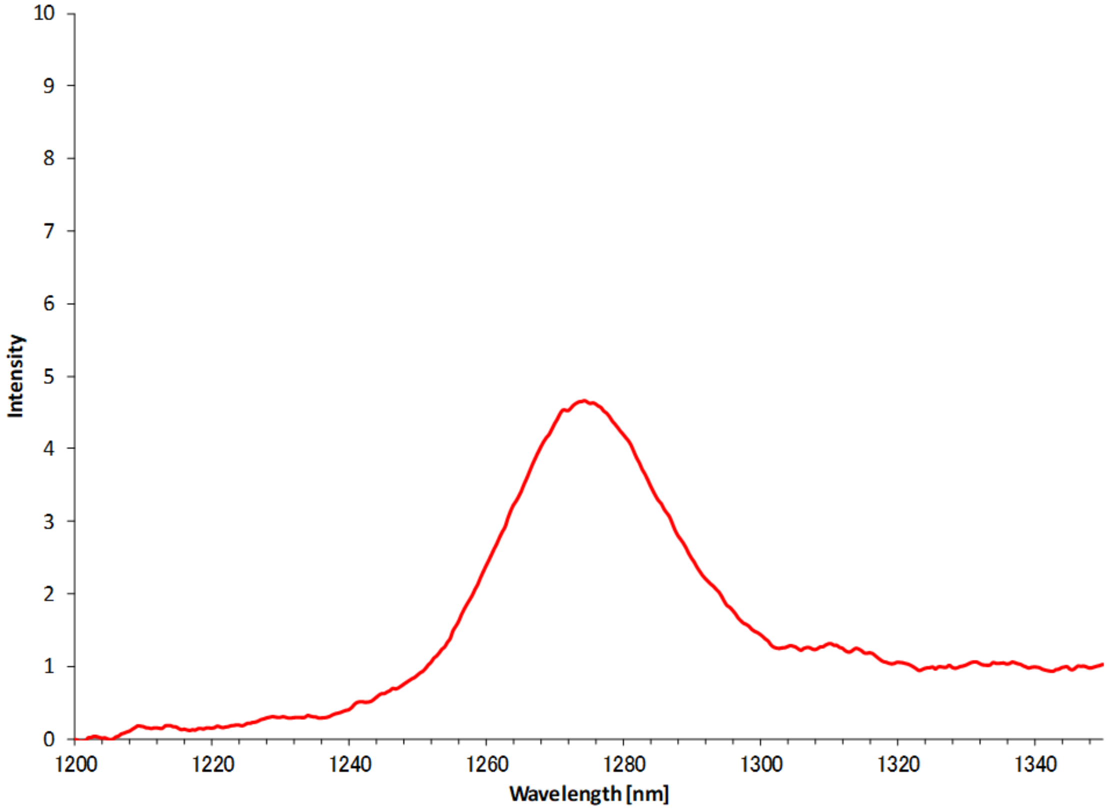

Figure S16. Emission spectra of singlet oxygen in the presence of **3** in toluene.

8. Reference

1. Gaussian 16, Revision C.01, Frisch, M. J., Trucks, G. W., Schlegel, H. B., Scuseria, G. E., Robb, M. A., Cheeseman, J. R., Scalmani, G., Barone, V., Petersson, G. A., Nakatsuji, H., Li, X., Caricato, M., Marenich, A. V., Bloino, J., Janesko, B. G., Gomperts, R., Mennucci, B., Hratchian, H. P., Ortiz, J. V., Izmaylov, A. F., Sonnenberg, J. L., Williams-Young, D., Ding, F., Lipparini, F., Egidi, F., Goings, J., Peng, B., Petrone, A., Henderson, T., Ranasinghe, D., Zakrzewski, V. G., Gao, J., Rega, N., Zheng, G., Liang, W., Hada, M., Ehara, M., Toyota, K., Fukuda, R., Hasegawa, J., Ishida, M., Nakajima, T., Honda, Y., Kitao, O., Nakai, H., Vreven, T., Throssell, K., Montgomery, J. A. Jr., Peralta, J. E., Ogliaro, F., Bearpark, M. J., Heyd, J. J., Brothers, E. N., Kudin, K. N., Staroverov, V. N., Keith, T. A., Kobayashi, R., Normand, J., Raghavachari, K., Rendell, A. P., Burant, J. C., Iyengar, S. S., Tomasi, J., Cossi, M., Millam, J. M., Klene, M., Adamo, C., Cammi, R., Ochterski, J. W., Martin, R. L., Morokuma, K., Farkas, O., Foresman, J. B. & Fox, D. J. Gaussian, Inc., Wallingford CT, **2019**.
